# Methyl Ethyl Ketone-Related Loss of Matrix With Nail Onycholysis and Pterygium (ME KLMNOP): Case Report of a New Etiology for Onycholysis and Pterygium

**DOI:** 10.7759/cureus.11597

**Published:** 2020-11-20

**Authors:** Parnia Forouzan, Philip R Cohen

**Affiliations:** 1 Dermatology, McGovern Medical School, University of Texas Health Science Center at Houston, Houston, USA; 2 Dermatology, San Diego Family Dermatology, National City, USA

**Keywords:** butanone, ethyl, ketone, loss, matrix, methyl, nail, onychodystrophy, onycholysis, pterygium

## Abstract

Methyl ethyl ketone is an organic solvent commonly used in adhesives and paints. Overexposure to methyl ethyl ketone can irritate the central nervous system, eyes, and respiratory system. When in direct contact with skin, methyl ethyl ketone can lead to dryness and cracking of the skin. Forty years ago, methyl ethyl ketone was used in the Navy to degrease and remove paint from planes and naval equipment. A 57-year-old Navy veteran presented with an absence of all fingernails and thumbnails as well as pterygium formation on his digits; however, his toenails were normal. Additional history revealed that his unprotected hands were regularly exposed to methyl ethyl ketone for three years. His nails shed and stopped growing after one year of this work; subsequently, pterygiums developed. We postulate that exposure to methyl ethyl ketone may result in chemical destruction of the nail matrix in a similar manner to phenol or sodium hydroxide. We introduce the following acronym that describes not only the etiology but also the manifestations of Methyl Ethyl Ketone-related Loss of Matrix with Nail Onycholysis and Pterygium (ME KLMNOP).

## Introduction

Methyl ethyl ketone, also referred to as 2-Butanone, is an organic solvent. It is a colorless, volatile liquid with an acetone-like odor. Methyl ethyl ketone can be found in natural products such as fruits and vegetables but is also manufactured for use in cleaning products, glues, and paints. Items containing methyl ethyl ketone can be purchased at hardware stores or online. Individuals employed in manufacturing, painting, or industrial work may be at greater risk for occupational exposure to methyl ethyl ketone [1].

Onychodystrophy is a descriptive term for an abnormal nail morphology. It can be congenital or acquired. Acquired onychodystrophy can be endogenous (disease-related) or exogenous (secondary due to etiologies such as trauma or chemical exposure) [2].

We report a patient who experienced chronic and persistent exposure to methyl ethyl ketone for three years. He developed an onychodystrophy characterized by nail matrix loss that resulted in onycholysis and subsequent pterygium formation. We introduce the following descriptive nomenclature for this nail dystrophy: Methyl Ethyl Ketone-related Loss of Matrix with Nail Onycholysis and Pterygium (ME KLMNOP).

## Case presentation

**Figure 1:**
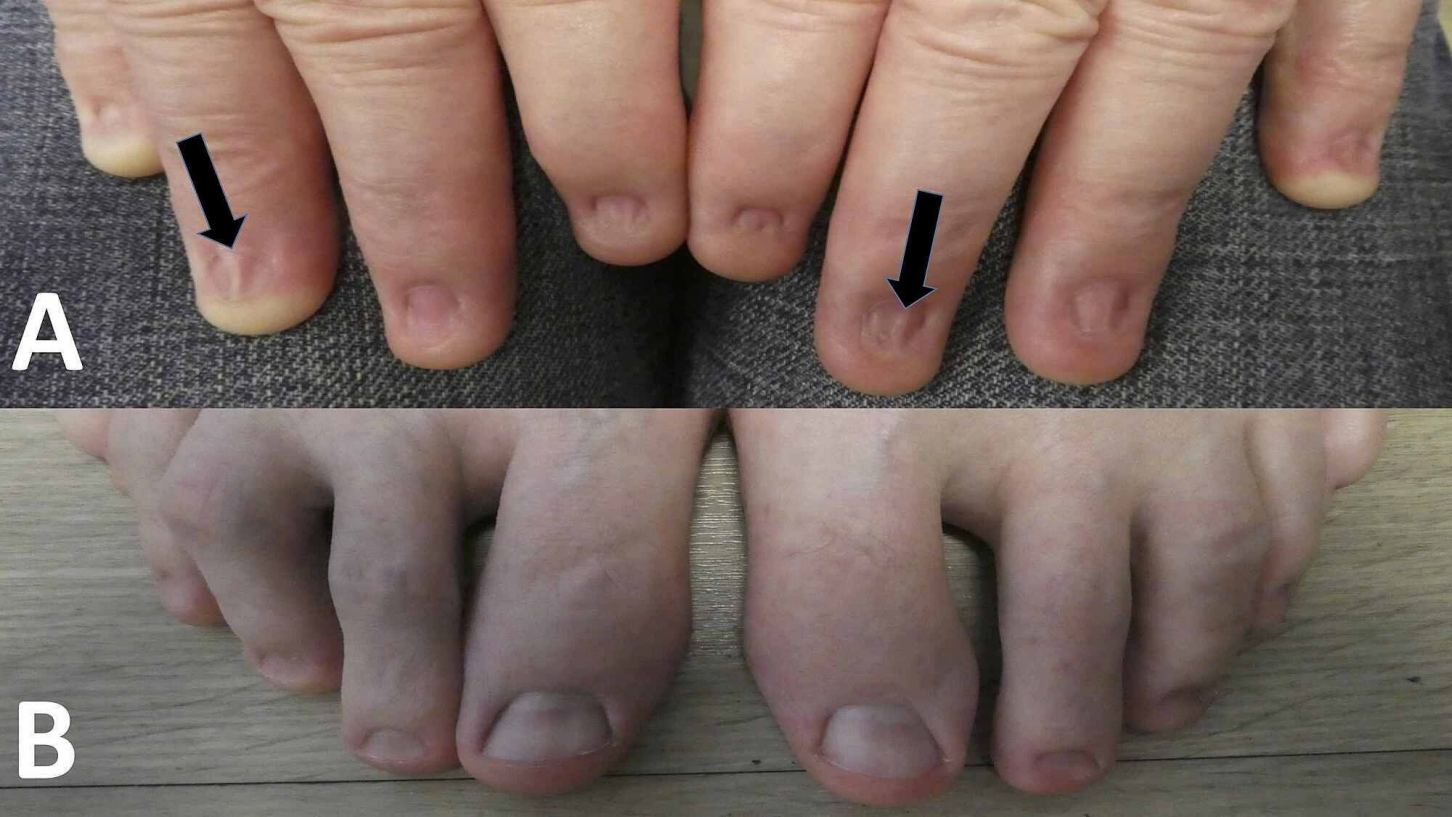
Clinical presentation of fingernails 40 years after repeated exposure to methyl ethyl ketone compared with the patient’s normal toenails The hands (A) and feet (B) of a 57-year-old man after repeated exposure of his unprotected hands to methyl ethyl ketone. There is an absence of all nails on both of his hands; also, many of the fingers have a scar that extends from the proximal nail fold onto the nail bed (black arrows).

**Figure 2:**
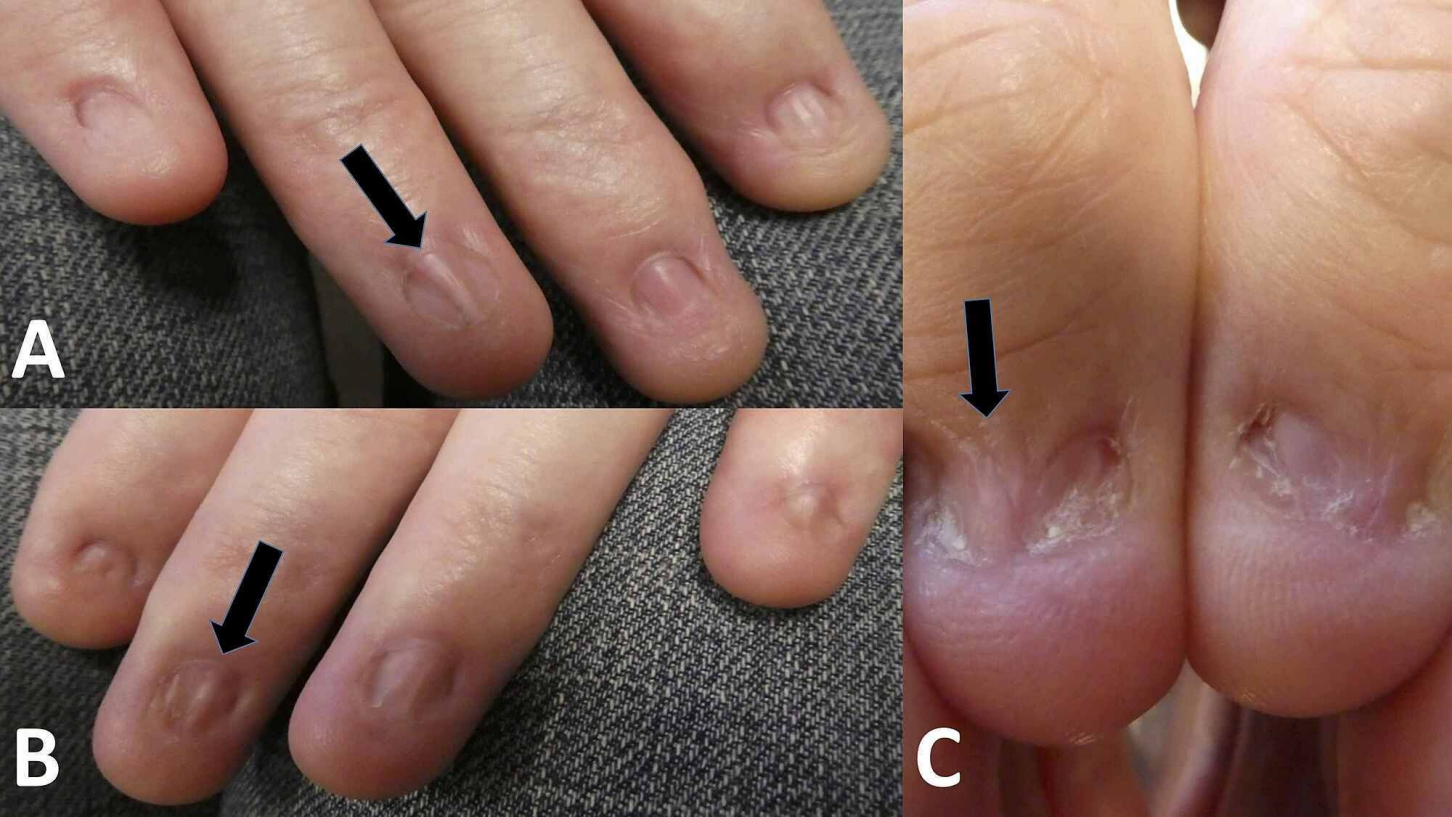
Onycholysis and pterygium formation on fingernails There is loss of all fingernails (A and B) and thumbnails (C) with subsequent pterygium formation (black arrows) in a Navy veteran after repeated hand exposure to methyl ethyl ketone.

A 57-year-old Caucasian man presented for a total body skin check. Multiple benign pigmented lesions were observed. However, examination of his hands revealed an absence of all fingernails and thumbnails; in contrast, his toenails were intact and normal in appearance (Figure 1). Pterygium, extending from the proximal nail fold to the nail bed, was also observed on multiple digits of his hands (Figure 2).

Additional history revealed that he entered the Navy 40 years ago. For three years, his work involved using methyl ethyl ketone to strip off adhesives, grease, and paint from planes and helmets. His digits would regularly be exposed to methyl ethyl ketone for 30 to 60 minutes several days per week approximately every other month.

The man - without hand protection such as gloves - would dip rags into small containers filled with methyl ethyl ketone; the rags were used to clean objects. After using the methyl ethyl ketone, his hands would burn. After washing his hands with soap and water, a tingling sensation transiently remained on his hands.

His nails initially turned white and then became flaky; therefore, he would file his nails flat. His nails stopped growing and then shed after one year of exposure to methyl ethyl ketone. The finger and thumb nails did not subsequently reappear. He also mentioned that similar nail changes were observed in other men who participated in the same naval work.

## Discussion

Methyl ethyl ketone is commonly used as a solvent in paints and coatings. It was also used in the 1980s by Navy personnel for removing paint and grease from equipment and planes. At the time, hand protection such as gloves was not used during exposure to this agent. Methyl ethyl ketone has subsequently been substituted with other agents [1].

Methyl ethyl ketone (C_4_H_8_O) is a flammable liquid and hazardous to the central nervous system, respiratory system, and skin. Workplace airborne limits of methyl ethyl ketone set by the Occupational Safety and Health Administration (OSHA) are 200 parts per million over a period of eight hours. If in direct contact with skin, overexposure can occur at the legal airborne limits [1].

Methyl ethyl ketone can severely irritate the eyes, nose, and throat. Contact with the vapor of methyl ethyl ketone may dry out these surfaces, leading to irritation. This can lead to permanent damage. In addition, with repeated or high exposure, this solvent causes dizziness, nausea, and damage to the nervous system [1].

When methyl ethyl ketone is in direct contact with the skin, it can lead to a rash or burning sensation. The mechanism for these adverse cutaneous reactions remains to be established; however, we speculate that they could be secondary to either an irritant contact dermatitis or cutaneous nerve damage or both. Longer exposures can dry out the skin. However, the effect of methyl ethyl ketone on nails that were observed in our patient has not been previously described [1].

The nail matrix is the growth center of the nail; the nail plate initiates from the nail matrix. Nail matrix injury can be exogenous or endogenous. Injury to the nail matrix results in abnormalities of the nail plate and can lead to shedding of the nail plate.

Exogenous causes of nail matrix injury such as trauma can result in brittle nails and nail splitting. Conditions such as alopecia areata, arteriosclerosis, lichen planus, peripheral arterial disease, polycythemia vera, and psoriasis can also damage the nail matrix; inflammation associated with some of these conditions may damage the nail matrix with subsequent nail thinning or deep pitting of the nail plate. In vascular diseases, decreased oxygenation and nutrient delivery to the nail matrix can cause brittle, deformed, or slow-growing nail plates [3].

Destruction of the nail matrix can be used for the permanent elimination of the nail plate. This can be done surgically. Alternatively, 88% phenol (C_6_H_5_OH) applied for four minutes or 10% sodium hydroxide applied for one minute can be used for chemical ablation of the nail matrix [4,5].

Phenol denatures and coagulates proteins leading to necrosis of the nail matrix which prevents regrowth of the nail [5]. Alternatively, sodium hydroxide leads to liquefaction necrosis of the nail matrix; this method of nail matrix ablation has been associated with faster healing compared to phenol matrixectomy [6]. We postulate that methyl ethyl ketone may behave in a similar pathophysiological manner to either phenol or sodium hydroxide, resulting in a chemical destruction of the nail matrix after repeated exposure.

Onycholysis is shedding of the nail plate. There is separation of the nail plate from the nail bed. Onycholysis is most commonly caused by physical trauma such as nail care practices or occupation-related events [2,7].

However phototoxic events and disease states have also been linked to onycholysis. Patients taking a tetracycline antibiotic, 5-fluorouracil, psoralen, or griseofulvin should be cautious of ultraviolet light exposure because of the risk for phototoxicity leading to onycholysis. Alopecia areata, amyloidosis, hypothyroidism, onychomycosis, pellagra, and psoriasis have also been associated with onycholysis [2,7].

When the nail matrix remains intact after onycholysis, a new nail can grow. If the nail matrix is ablated, a subsequent nail will not appear. In our patient, onycholysis occurred subsequent to the destruction of his nail matrix, and his nails did not reappear.

Pterygium of the distal digit usually refers to a scar that extends from the proximal nail fold onto the nail bed. However, a distal pterygium, referred to as pterygium inversus ungium, describes the fusing of the distal nail plate to the adjacent nail bed. Pterygium inversus ungium can be associated with dermatomyositis, Raynaud’s phenomenon, systemic lupus erythematosus, and trauma [2].

Pterygium may occur following trauma to the nail. Alternatively, it can be associated with certain conditions such as lichen planus and scleroderma. Our patient developed pterygiums on multiple hand digits after the loss of his fingernails and thumbnails.

## Conclusions

Chronic and repeated exposure to methyl ethyl ketone can result in severe onychodystrophy. Our patient’s nail abnormality included nail matrix destruction, nail plate shedding, and subsequent pterygiums. Similar nail changes also occurred on the hand digits of other men in the Navy after comparable exposures to methyl ethyl ketone. Therefore, methyl ethyl ketone can be added to the etiologies for onycholysis and pterygium formation. We postulate this organic solvent caused chemical ablation of the nail matrix similar to that observed after exposure of the nail matrix to phenol or sodium hydroxide. We suggest an acronym that describes not only the etiology but also the manifestations of Methyl Ethyl Ketone-related Loss of Matrix with Nail Onycholysis and Pterygium: ME KLMNOP.
